# The serum 25-hydroxyvitamin D levels and hip fracture risk: a meta-analysis of prospective cohort studies

**DOI:** 10.18632/oncotarget.16337

**Published:** 2017-03-17

**Authors:** Qing-Bo Lv, Xiang Gao, Xiang Liu, Zhen-Xuan Shao, Qian-Hui Xu, Li Tang, Yong-Long Chi, Ai-Min Wu

**Affiliations:** ^1^ Department of Orthopedics, Bone Research Institute, The Second Affiliated Hospital and Yuying Children's Hospital of Wenzhou Medical University, Second Medical School of Wenzhou Medical University, Wenzhou, Zhejiang, China

**Keywords:** serum 25-hydroxyvitamin D, hip fracture, meta-analysis, dose-response

## Abstract

Hip fracture has increasingly become a social and economic burden. The relationship between serum 25-hydroxyvitamin D levels and the risk of hip fracture reported by previous studies remains controversial. We searched Pubmed and Embase to identify studies reporting the relationship between serum 25-hydroxyvitamin D levels and risk of hip fracture. Fifteen prospective cohort studies with a total of 51239 participants and 3386 hip fracture cases were included. By pooling the Relative Risk of the lowest vs. the highest categories indicated that lower levels of serum 25-hydroxyvitamin D were more likely to be a risk factor for hip fracture with adjusted Relative Risk (95%Confidence Interval) of 1.58 (1.41, 1.77). Subgroup meta-analysis examining the stability of the primary results achieved the same results. A dose-response meta-analysis showed that the risk of hip fracture was a descending curve below the line of RR=1. The descending trend was obvious when serum 25-hydroxyvitamin D levels were less than 60 nmol/L and was flat when serum 25-hydroxyvitamin D levels were more than 60 nmol/L. We found that individuals with low levels of serum 25-hydroxyvitamin D have an increased risk of hip fracture, and this effect was evident when the serum 25-hydroxyvitamin D levels were less than 60 nmol/L.

## INTRODUCTION

Hip fracture as a serious consequence of osteoporosis is becoming an important health problem, and an increasing social and economic burden [1–3], and hip fractures are expected to receive more attention over the coming years [4–6]. Cenzer et al [7] reported that about 27% hip fracture patients dead during the 1-year follow up. Multiple factors have been shown to be associated with the risk of hip fractures [8–11]. It is widely recognized that vitamin D plays an important role in skeletal health [12–15].

Serum 25-hydroxyvitamin D [25(OH)D] is widely recognized as the indicator of vitamin D [16]. Steingrimsdottir et al [17] found that serum 25(OH)D < 30 nmol had higher risk of hip fractures than the 25(OH)D with 50-75 nmol/L (Hazard ratios: 2.24 (95% CI 1.63, 3.09)). Similar result was found in study of Looker et al [18]. However, in study of Barbour et al [19], they found no evidence of an association between 25(OH)D and any non-spine fractures, and similar result was found in studies of Cummings et al [20] (25(OH)D less than 47.5 nmol/L (19 ng/ml) with RR: 1.2 (0.7-1.9)), Chan et al [21] with (RR: 0.63 (0.15-2.62)), and de Boer et al [22] with (RR: 1.34 (0.97-1.84)).

Therefore, the effect of serum 25(OH)D concentrations on hip fracture remains uncertain, and we conducted this meta-analysis for the purpose of determining the association between serum 25(OH)D concentrations and the risk of hip fracture. Moreover, which is optimal serum 25(OH)D concentration? And will the sex, age, geographic region or study design (cohort or nested case-cohort) influence the outcomes? Dose-response and subgroup analysis (sex, age, geographic region or study design) were performed to help us understand more about serum 25(OH)D levels and their relationship to the risk of hip fracture.

## RESULTS

### Literature search

**Figure 1:**
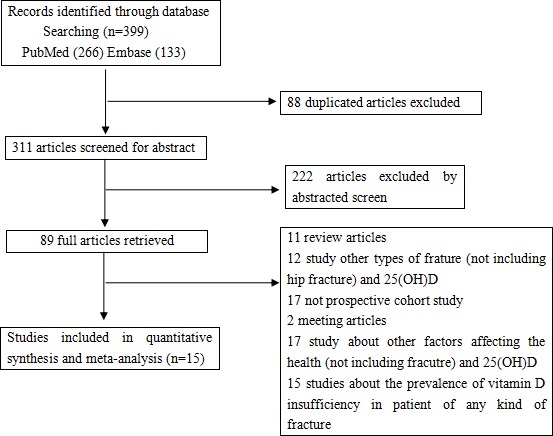
The selection of literature for included studies

The detailed steps of our literature selection are shown in Figure 1. A total of 399 potential records were identified from the databases, including 88 duplicate articles, 311 articles leaved after duplications deleted; then, 222 papers were excluded by screening the abstracts, and 89 full articles were retrieved. There were 74 articles excluded, including 11 review articles, 12 studies that examined other types of fractures and 25(OH)D, 17 non-prospective cohort studies, 2 meeting articles, 17 articles about other factors affecting the bone health (not including fracture) and 25(OH)D, and 15 studies research the prevalence of vitamin D insufficiency in patient of any kind of fracture (didn't have hip fracture data). Overall, Fifteen prospective cohort studies [17–31] that referred to the relationship between serum 25(OH)D levels and hip fractures were included.

### Study characteristics

The characteristics of the included studies are presented in Table 1. A total of 51239 participants were included in our meta-analysis, of whom 3386 had a hip fracture. Fourteen of the fifteen studies were conducted during the period from 2007 to 2016, and there was only one article that was conducted in 1998. Of these fifteen studies, there were eleven cohort studies and four nested case-cohort studies; five of the studies were conducted in Europe and eight were conducted in the United States, one was conducted in the HongKong and one was conducted in the New Zealand. These studies met the criteria for high quality (7 to 9 stars). Most studies provided risk estimates that were adjusted for age (14 studies), BMI (10 studies), gender (8 studies), drinking (7 studies), smoking (10 studies), physical activity (7 studies) and weight (4 studies) (Table 1).

**Table 1:** Characteristics of Prospective Studies on Serum 25-Hydroxyvitamin D and Hip Fracture

Source	Study Type	No. of participants	Location/ Period	Gender	Age (years)	No. of casesa	Measure/Range of concentrations	Study Qualityb	Adjustment for Covariatesc
Steingrimsdottir et al 2014	Cohort study	5461	Iceland 2002-2009	F:3125 M:2346	66–96	261HF	Q1<30 nmol/L 30 nmol/L≤Q2<50 nmol/L 50 nmol/L≤Q3<75 nmol/L Q4≥75 nmol/L (Immune based)	9	Age, sex, body mass index, height, smoking, alcohol intake and season,physical activity.
Khaw et al 2014	Cohort study	14641	United Kingdom 1997-2012	F:8155 M:6486	42–82	198HF	Q1<30 nmol/L 30 nmol/L≤Q2<50 nmol/L 50 nmol/L≤Q3<70 nmol/L 70 nmol/L≤Q4 <90 nmol/L Q5≥90 nmol/L (Chromatography based)	9	Age, sex, month, BMI, physical activity, smoking, alcohol, vitamin C, diabetes, history of cardiovascular disease, history of cancer, social class, and education .
Barbour et al 2012	Cohort study	2640	United States 1997-2009	F:1291 M:1349	70-79	84 HF	Q1≤44.45 nmol/L 44.48 nmol/L≤Q2≤60.9 nmol/L 60.93 nmol/L≤Q3≤79.85 nmol/L Q4> 79.85nmol/L (Immune based)	9	Age, gender, race, education level, season of blood draw, BMI, current drinking, fracture after age 45 and clinical comorbidity index.
Robinson-Cohen et al 2011	Cohort study	2294	United States 1989–2009	F:1600 M:694	≥65	244HF	Q1<37.5nmol/L Q2≥37.5nmol/L (Chromatography based)	8	Age, race, sex, clinic site, a season, education, smoking status (never smoker, former smoker, or current, smoker), alcohol use (any vs. none), diabetes status (normal, impaired fasting glucose, or diabetes), body mass index, self-reported health status, physical activity level, oral steroid use, estrogen use, thiamine and loop diuretic use, serum cystatin C level, and calcium supplement use.
Looker et al 2008	Cohort study	1917	United States 1991–2000	F:986 M:931	≥65	156HF	0nmol/L≤Q1≤42.9 nmol/L 43nmol/L≤Q2≤61 nmol/L 61.1nmol/L≤Q3<82.5 nmol/L Q4 ≥82.5nmol/L (Immune based)	9	Age, sex, femoral neck BMD, BMI, previous fracture, dietary calcium, kilocalories, and weight loss from maximum.
Holvik et al 2013	Case-cohort study	2526	Norway 1994–2001	F:1819 M:707	65-79	1175HF	4.5 nmol/L≤Q1≤42.1 nmol/L 42.2 nmol/L≤Q2<53.5 nmol/L 53.5 nmol/L≤Q3≤67.8nmol/L 67.9 nmol/L≤Q4≤250.0 nmol/L (Mass spectrometry)	9	Age, gender, study center, BMI, and month of blood sample.
Cauley et al 2010	Case-cohort study	1665	United States 2000-2002	M:1665	≥65	81HF	7.83 nmol/L≤Q1<47.5 nmol/L 47.5 nmol/L≤Q2<62.75 nmol/L 62.75 nmol/L≤Q3<74.75 nmol/L Q4≥74.75 nmol/L (Chromatography based)	8	Age, race, clinic, season of blood draw, physical activity, weight, and height.
Cauley et al 2008	Case-cohort study	800	United States 1994-2004	F:800	50-79	400HF	9.23 nmol/L≤Q1<47.6 nmol/L 47.6nmol/L≤Q2<60.2 nmol/L 60.2 nmol/L≤Q3<70.7nmol/L 70.7 nmol/L≤Q4<121.5 nmol/L (Immune based)	9	Age, body mass index, parental history of hip fracture, history of fracture, smoking, alcohol use, and total calcium intake, oral corticosteroid use and geographic region.
Cummings et al 1998	Case-cohort study	476	United States 1986-1988	F:476	≥65	133HF	Q1<47.5 nmol/L Q2≥47.5 nmol/L (Immune based)	8	Age and weight
Bolland et al 2010	Cohort study	1471	New Zealand 1998-2003	F:1471	>50	22HF	Q1<50 nmol/L Q2≥50 nmol/L (Immune based)	9	Treatment allocation (calcium or placebo) and baseline age, body weight, and smoking status.
Melhus et al 2010	Cohort study	1194	Sweden	M:1194	>50	73HF	Q1<50 nmol/L Q2≥50 nmol/L (Chromatography based)	9	Weight, height, age, cystatin C, calcium intake, season, physical activity, smoking, diabetes mellitus, other endocrine disease, hematological diseases, dermatoses, infectious disease, musculoskeletal disease, psychiatric disease, neurological disease, respiratory disease, kidney or urinary disease, gastrointestinal disease.
Chan et al 2011	Cohort study	712	HongKong	M:712	≥65	24HF	Q1≤63 nmol/L 63 nmol/L<Q2≤76 nmol/L 76 nmol/L<Q3≤91 nmol/L Q4>91 nmol/L (Immune based)	9	Age, BMI, education, PASE, DQI, smoking status, and alcohol use
Buchebner et al 2014	Cohort study	1044	Sweden	F:1044	≥75	130HF	Q1<50 nmol/L 50 nmol/L≤Q2≤75 nmol/L Q3>75 nmol/L (Chromatography based)	7	smoking, bisphosphonate use, and physical activity level
de Boer et al 2012	Cohort study	1621	United States 1992–2006	F:1130 M:491	≥65	137HF	Q1<50 nmol/L Q2≥50 nmol/L	9	Age, sex, clinical site, smoking, body mass index, and physical activity.
Takiar et al 2015	Cohort study	12781	United States 1990-1992	F:7196 M:5585	Mean 57	267HF	Q1<50 nmol/L Q2≥50 nmol/L	9	Age, sex, and race/cente, potentially confounding variables of education, annual household income, physical activity, smoking status, alcohol drinking status, body mass index, waist-to-hip ratio, diabetes, systolic and diastolic blood pressure, use of hypertension medication, total and HDL cholesterol, estimated glomerular filtration rate, thiazide diuretic usage, and hormone replacement therapy, calcium, phosphate, and PTH levels.

a: HF: Hip fracture.

b: Study quality was judged based on the Newcastle-Ottawa Scale (range, 1-9 stars).

c: BMI: body mass index; BMD: Bone mineral density.

### Serum 25(OH)D levels and hip fracture risk

**Figure 2:**
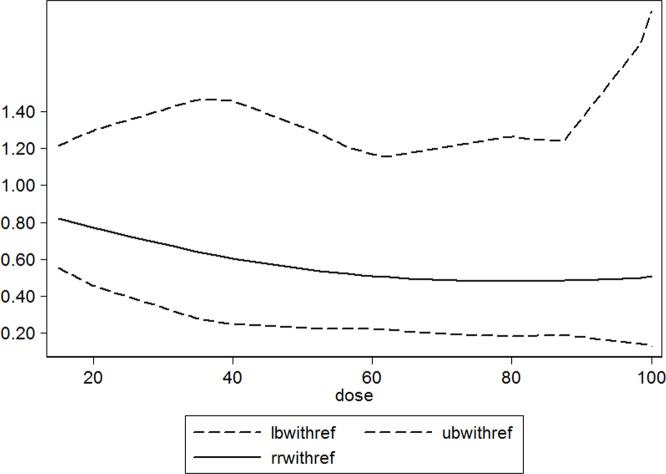
Adjusted Relative Risks of hip fracture for the lowest *vs*. **highest categories of serum 25(OH)D levels**.

There were thirteen studies that referred to the relationship between serum 25(OH)D levels and the risk of hip fracture. The results of the meta-analysis are shown in Figure 2. The pooled adjusted RRs of hip fracture for the lowest *versus* highest categories of serum 25(OH)D concentrations were 1.58 (1.41, 1.77) without significant heterogeneity (I^2^ = 16.8%, *P* = 0.265), the meta-regression of year, age, follow-up term, study-type, gender and location showed no heterogeneity was found (all of the *P* > 0.10, Supplementary Figure S1). Additionally, there was no indication of significant publication bias *via* Egger's test (*P* = 0.427).

**Figure 3:**
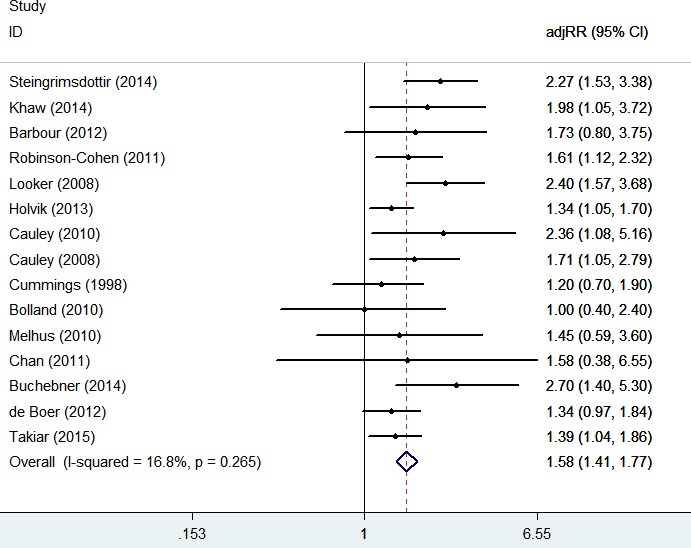
Dose-response relationship between serum 25(OH)D and relative risk of hip fracture Solid line represents adjusted relative risk and dotted lines represent the 95% confidence intervals for the fitted trend. Adj.RR of hip fracture is a descending curve below the line of RR = 1. The descending trend was obvious when serum 25(OH)D level was less than 60 nmol/L, and flat when serum 25(OH)D was higher than 60 nmol/L; there was no significant linear association between the serum 25(OH)D levels and the risk of hip fracture (*P* = 0.110 for non-linearity).

### Subgroup meta-analysis

The results of the subgroup meta-analysis are shown in the Table 2. We found that low levels of serum 25(OH)D increased the risk of hip fracture when it was defined by gender (male: 1.86 (1.36 to 2.56); female: 1.45 (1.20 to 1.70)), age (≥ 65 years: 1.61 (1.41 to 1.84); ≥ 42 years: 1.49 (1.19 to 1.85)), location (Europe: 1.64 (1.37 to 1.97); USA: 1.56 (1.34 to 1.80)), study type (cohort: 1.67 (1.45 to 1.93); nested case-cohort: 1.42 (1.17 to 1.72)), and duration of follow-up (< 7 years: 1.89 (1.52 to 2.35); ≥ 7 years: 1.48 (1.29 to 1.69)).

**Table 2:** Subgroup analyses for 25(OH)D and hip fracture

	No. of reports	Relative Risk (95CI%)	*P* for heterogeneity	*I*^2^	*P* for test
Sex					
Male	5	1.86 (1.36 to 2.56)	0.695	0.0	0.000
Female	6	1.45 (1.20 to 1.75)	0.181	34.1	0.000
Age					
≥65 years	10	1.61 (1.41 to 1.84)	0.110	37.3	0.000
≥42years	5	1.49 (1.19 to 1.85)	0.722	0	0.000
Location					
Europe	5	1.64 (1.37 to 1.97)	0.096	49.2	0.000
USA	8	1.56 (1.34 to 1.80)	0.355	9.7	0.000
Study type					
Cohort	11	1.67 (1.45 to 1.93)	0.277	17.5	0.000
Case-cohort	4	1.42 (1.17 to 1.72)	0.418	0.0	0.000
Duration of follow-up					
<7 years	7	1.89 (1.52 to 2.35)	0.271	20.8	0.000
≥7 years	6	1.48 (1.29 to 1.69)	0.575	0.0	0.000

### Dose-response meta-analysis

Four studies [17, 21, 24, 26] of serum 25(OH)D concentration and risk of hip fracture met the dose-response meta-analysis criteria. The results of generalized least-squares regression for serum 25(OH)D levels and the adj.RR of hip fracture are shown in the Figure 3 as a descending curve below the line of RR = 1. The descending trend was obvious when the serum 25(OH)D level was below 60 nmol/L and flattened when the serum 25(OH)D level was higher than 60 nmol/L; there was no significant linear association between the serum 25(OH)D levels and the risk of hip fracture (*P* = 0.110 for non-linearity, Figure 3).

## DISCUSSION

Hip fracture has been worldwide burden [32] and the association of low levels of serum 25(OH)D and risk of fracture among older adults was unclear [18, 19, 23, 25, 27, 33]. A previous meta-analysis based on randomized controlled trials and observational studies [34] showed that neither higher nor lower doses of vitamin D supplementation led to an increased risk of hip fracture; it found that the serum 25(OH)D levels in the 17 identified case-control studies were 33% lower in the case groups compared to the control groups [34].

We found that the adjusted relative risk (adj.RR) of low levels of 25(OH)D has a statistically significant 58% increased risk of hip fracture. All of the subgroup meta-analyses of different genders, age ranges, locations, study types, and durations of follow up supported the result that lower serum 25(OH)D levels significantly increased the risk of hip fracture.

Serum 25(OH)D is the indicator of vitamin D. Therefore, low levels of serum 25(OH)D may be associated with bone loss, lower bone mineral density (BMD) and higher bone turnover [35], which may cause a high risk of fracture. Some other indirect factors may increase the risk of hip fracture, including muscle strength, balance and intake of nutrients; the deficiency of vitamin D may decrease muscle strength and balance, and may also be combined with deficiencies of other nutrient [36–38]. It was also reported that vitamin D deficiency may increase the risk of falls, which then may increase hip fracture risk [39, 40]. Finally, low levels of serum 25(OH)D may be connected with poor health status, which also causes an increase in the risk of hip fracture [41].

The guidelines published by the Institute of Medicine (IOM) recommend that serum 25(OH)D levels ≥ 50 nmol/L(≥ 20 ng/ml) can benefit skeletal health [12]. In this study, the dose-response meta-analysis showed a descending curve below the line of RR = 1, which was consistent with the results of the forest plot in Figure 2; however, no significant curvilinear association between serum 25(OH)D levels and the risk of hip fracture was found. Additionally, using the RR curve in Figure 3, we found that the curve descended before the level of 60 nmol/L, then the curve trend became flat; therefore, when serum 25(OH)D levels are < 60 nmol/L, increasing serum 25(OH)D levels may significantly decrease the risk of hip fracture, and after reaching 60 nmol/L, increasing serum 25(OH)D levels will not decrease the risk of hip fracture. The transitional serum 25(OH)D level in this meta-analysis (60 nmol/L) was 10 nmol/L higher than that recommended by the Institute of Medicine (IOM) (≥ 50 nmol/L) [12].

### Strengths of this study

The strength of this meta-analysis is that our quantitative assessment is based on prospective cohort studies, which can overcome the weaknesses of recall and selection bias in case-control studies and provide more data on hip fracture risk within the population. To the best of our knowledge, this is the first systematic review and meta-analysis of the relationship between serum 25(OH)D levels and the risk of hip fracture based on prospective cohort studies. Moreover, we performed subgroup analyses by gender, age, location, study design and follow-up term, and the results were consistent to the overall result. We also performed dose-response assessments of the relationship between serum 25(OH)D levels and the risk of hip fracture. Finally, all of the included studies had high quality assessment scores (7-9 stars, nine-star Newcastle-Ottawa scale) and long durations of follow-up, with a large population of 51239 participants totally.

### Limitations of this study

There are still some limitations of our meta-analysis. Firstly, not all of the studies met the requirement of dose-response meta-analysis; only four of included studies were pooled for dose-response meta-analysis. Secondly, There were some different 25(OH)D assays exist, in general, they can be grouped into 2 different categories: immune based and chromatography based [42]. According to the current views, the intra-assay variation may reach 0-10%, while the inter-assay variation may reach 0-25% (at lower serum levels) [43, 44]. Therefore, the variation is existed, and can't be avoided, we should be kept in mind when following an individual patient over time [44]. Thirdly, some factors that vitamin D measurements in blood samples drawn from fracture patients in the acute phase may be influenced by the trauma and surgery itself-such as by inflammation [45] or hemodilution caused by fluid supply [46].

## CONCLUSIONS AND CLINICAL IMPLICATION

Individuals with low levels of serum 25(OH)D have an increased risk of hip fracture, and this effect was evident when the serum 25(OH)D level was less than 60 nmol/L, and disappeared when the serum 25(OH)D level was more than 60 nmol/L.

## MATERIALS AND METHODS

The present study was completed according to the preferred reporting items for systematic review and meta-analyses (PRISMA) statement (Checklist S1).

### Search strategy

We systematically searched PubMed and EMBASE on September 15, 2016 using the following keywords: 1) 25-hydroxyvitamin D, 25(OH)D, or vitamin D; 2) hip fractures or hip fracture; 3) cohort study, cohort studies, prospective study, prospective studies, or longitudinal study. We conducted the searches without language and publication year restrictions. In addition, we manually scanned the related articles and used the “related article” function for additional searches to avoid initial misses.

### Selection criteria

The criteria for inclusion in this meta-analysis were as follows: (1) the study was designed as a prospective cohort study; (2) exposure of interest is 25(OH)D; (3) primary outcome of interest is hip fracture; and (4) relative risk (RR) and the corresponding 95% confidence interval (CI) were reported or could be calculated using the data reported. If the data were overlapped or duplicated by multiple publications, the study with the larger number of cases was included

And the criteria of exclusion were: (1) the retrospective study; (2) No data of 25(OH)D and hip fracture was described; (2) meeting abstract that couldn't found the full text; (4) the RR and 95%CI were not reported or couldn't be calculated using the reported data.

Two authors (QBL and XG) independently scanned the potential studies to determine which studies were eligible. Disagreements were discussed first and resolved by a third independent author (AMW).

### Data extraction

Three investigators (XL, QHX and ZXS) independently extracted data and the other author (YLC) cross-checked the data for consistency. Differences and disagreements were resolved by consensus. The data were recorded using a standard data extraction form, including the first author's last name, publication year, country where the study was performed, study period, sample size (cases and controls or cohort size), gender and age of participants, measure and range of exposure, variables adjusted for analysis, and RR estimates with corresponding 95% CI for each category of 25(OH)D. If there were more RRs for different potential confounders, we extracted the RRs that reflected the greatest degree of control for potential confounders. If necessary, we contacted the authors of the primary studies for additional information. The nine-star Newcastle-Ottawa scale was used to assess the study quality [47, 48].

### Statistical analysis

First, RR estimates were combined using a fixed-effects model as a common measure of the relationship between serum 25(OD)D levels and the risk of hip fracture across studies. To obtain a more accurate result, we pooled the RRs and 95%CIs of the lowest *vs*. the highest categories for synthesis to determine the risk of hip fracture with low 25(OH)D concentrations [49–51].

Second, we conducted a subgroup meta-analysis to examine the significance of the difference in RRs by different subgroups, including gender (male and female), age (≥ 65 years and ≥ 42 years), location (Europe and USA), study type (cohort study and case cohort study) and the duration of follow-up (< 7 years and ≥ 7 years). And the covariates (year, age, follow-up term, study-type, gender and location) meta-regression was performed to observe the potential heterogeneity.

Third, to examine the association between 25(OH)D levels and the risk of hip fractures, a dose-response meta-analysis was fitted using 4 knots at the following percentiles of distribution: 5%, 35%, 65% and 95% of the distribution [52]. We used the random-effects model reported by Greenland and Longnecker [53] and Orsini et al [54] to conduct a dose-response meta-analysis of 25(OH)D levels. For consistency within the included studies, 25(OH)D concentrations are reported in “nmol/L”. Concentrations reported in “ng/ml” by some studies were converted using a conversion factor (1 ng/ml = 2.5 nmol/L).

Finally, statistical heterogeneity between studies was quantified by using Cochran's Q and the I^2^ statistic [55]. To investigate the influence of the individual data on the pooled results, we performed a sensitivity analysis to assess the influence of a single study on the pooled RR estimate by removing one study at a time. We also performed the Egger's asymmetry regression test to evaluate potential publication bias [56]. All statistical tests were performed using STATA software (Version 12.0; Stata Corp, College Station, Texas).

## SUPPLEMENTARY MATERIALS FIGURES AND TABLES


