# Supplementary material for: The serum 25-hydroxyvitamin D levels and hip fracture risk: a meta-analysis of prospective cohort studies
Source: Oncotarget. 2017 Mar 17;8(24):39849–58. doi: 10.18632/oncotarget.16337 (PMC5503658; doi:10.18632/oncotarget.16337)
Supplement: Supplementary file 1 [file oncotarget-08-39849-s001.pdf]

The serum 25-hydroxyvitamin D levels and hip fracture risk: a meta-analysis of prospective cohort studies

Supplementary Material

Supplementary Figure S1: The covariate meta-regression of year, age, follow-up term, study-type, gender and location showed no heterogeneity was found (all of the P>0.10, see the red dash box)

lnrr	Coef.	Std. Err.	t	P>|t|	[95% Conf.	Interval]
year	.0380316	.1226795	0.31	0.769	-.2773261	.3533893
age	-.0825492	.656901	-0.13	0.905	-1.771167	1.606069
followup	-.3588411	.6932849	-0.52	0.627	-2.140987	1.423304
cohort	.1998024	.6754488	0.30	0.779	-1.536494	1.936099
femaleandmale	-.3920224	1.001775	-0.39	0.712	-2.967168	2.183123
male	-.1429906	1.404085	-0.10	0.923	-3.752305	3.466324
newzealand	-.4817315	3.608009	-0.13	0.899	-9.756413	8.79295
european	.5687632	3.073057	0.19	0.860	-7.330782	8.468309
usa	.5047373	3.070233	0.16	0.876	-7.387549	8.397023
_cons	-75.63766	245.3524	-0.31	0.770	-706.336	555.0607
